# Associations of Shift Work and Its Duration with Work-Related Injury among Electronics Factory Workers in South Korea

**DOI:** 10.3390/ijerph14111429

**Published:** 2017-11-21

**Authors:** Jia Ryu, Kyunghee Jung-Choi, Kyung-Hwa Choi, Ho-Jang Kwon, Chungwon Kang, Hyunjoo Kim

**Affiliations:** 1 Department of Occupational and Environmental Medicine, Ewha Womans University College of Medicine, Seoul 07985, Korea; jiyajiya000@gmail.com (J.R.); jungchoikh@gmail.com (K.J.-C.); 2 Taean Environmental Health Center, Taean 32148, Korea; rosach72@hanmail.net; 3 Department of Preventive Medicine, Dankook University College of Medicine, Cheonan 31116, Korea; hojangkwon@gmail.com; 4 Department of Occupational and Environmental Medicine, Ewha Womans University Mokdong Hospital, Seoul 07985, Korea; scmfer@hanmail.net

**Keywords:** shift work, shift work duration, work-related injuries, gender difference

## Abstract

This study aimed to explore the association between shift work and work-related injuries. We collected data on workers from an electronics factory. This cross-sectional study included 13,610 subjects, who were assessed based on a self-reported questionnaire about their shift work experiences, work-related injuries, and other covariates. Multiple logistic regression models were used to evaluate the associations between shift work and work-related injuries and were estimated using the odds ratio. We found that the current and past shift workers, compared to non-shift workers, were associated with a 2.7- and 1.7-fold higher risk of work-related injury. There was a dose-response relationship between shift work duration and work-related injury among current female shift workers. Shift work increased the risk of work-related injuries, and the impact could be different depending on gender.

## 1. Introduction

Industrialization brought about a change in standard of living. Electricity has helped change our society by providing power for labor- and time-saving devices. Shift work is a form of employment designed to support service or production for 24 h of the day [1]. This employment form has become indispensable in industrialized countries to maintain manufacturing industries, medical services, and transportation businesses [2]. Shift work is a common working schedule in many countries. Approximately 15–30% of workers are engaged in shift work in developed countries [3,4,5]. The overall prevalence rate of alternative shift work was reported to be 28.7% in the USA according to data from the 2010 National Health Interview Survey [6]. In South Korea, 11% of employees were shift workers performing night work, based on the 2010 survey of work conditions by the Korean Ministry of Employment and Labor [7]. Several studies have been conducted on the effects of shift work on health. It has been associated with various health problems such as sleep disorders, gastrointestinal diseases, metabolic disorders, and cardiovascular diseases [8]. Shift work increases not only the risk of these negative health effects but also that of work-related injuries [9,10,11].

According to official industrial disaster statistics, occupational injury including death accounts for about 62,000 per year in South Korea. In addition, the manufacturing industry has the third highest rate of occupational injuries following the industry of mining and construction [12]. The work-related injuries or accidents at the workplace not only cause an impairment of individuals’ health but also affect the productivity of the workplace. These work-related injuries are related not only to the flagship industries of the country, but also to the scale of the national economy [13]. Furthermore, the relevant articles reported that the occurrence of the work-related injuries almost certainly reflect the combined impact of a large number of factors: the structure of workplace such as multi-tier contracting [14], psychosocial factor [15], and human behavior [16]. Most occupational injuries are preventable. Therefore, injuries in the manufacturing sector by workers with shift work are a major concern in terms of safety and health in South Korea.

Most previous studies investigating the association between shift work and work-related injuries were performed on health care workers who had worked irregular rotating shift schedules [17,18,19,20,21] or were using national data that did not provide detailed information on shift work [10,11]. There are few studies on the manufacturing industry, but shift work is prevalent in industries such as the electronics industry, operating 24 h a day in the regular rotating shift work system [2,22]. More studies on various occupations and industries could contribute to expanding our knowledge on shift work and its consequences. Several limitations of the previous studies [17,18] are that these studies could not fully explore gender differences in the association between shift work and work-related injuries, mainly because their studies were conducted in women-dominant workplaces. In addition, the negative effect of shift work on work-related injuries is explained by fatigue [23] or mental stress faced by workers contributing their lack of concentration [17]. Circadian rhythm disruption could also contribute to the worsening of the human cognitive function [24]. However, it is uncertain whether these effects were due to acute or chronic change. This issue could be answered by exploring the association between the duration of shift work and work-related injuries.

Therefore, we investigated the association of shift work and its duration with work-related injuries, and we explored gender differences among electronics factory workers under the regular rotating shift work system in South Korea.

## 2. Materials and Methods

### 2.1. Study Subjects

We collected data on 21,969 workers from an electronics factory in South Korea. From 9 April to 21 May 2015, surveys were conducted through a self-report questionnaire. All workers provided written informed consent to be study participants. Of a total of 14,241 respondents (response rate of 64.8%), 13,610 were selected as the final research subjects, excluding 631 respondents who did not report work-related injuries in the previous year. The study protocol was approved by the Institutional Review Board of Korea National Open University (IRB approval number: ABN01-201502-11-02).

### 2.2. Major Variables

The structured questionnaire consisted of information about demographic characteristics, work-related characteristics, lifestyle, and health status. We selected the variables related to the association of shift work and work-related injuries. Other variables such as gender, age, education level, marital status, high-risk alcohol consumption (more than two times per week), smoking, safety health score, place of work, and type of work were used as potential confounders for adjusted analyses.

### 2.3. Shift Work

In the electronics factory, all shift workers worked as rotating shift workers in four teams with three 8 h shifts ensuring production 24 h a day, 7 days a week, 365 days a year. Each team worked for six days and had two days off. The factory was located in two cities, and the two factories had the same work schedule, differing only in work starting time. Factory B operates one hour earlier than Factory A. In other words, the shift workers worked the first six days in the morning shift (07:00–15:00), the second six days in the afternoon (15:00–23:00), and the third six days in the night (23:00–07:00). Two days between each six-day cycle are rest days. This system has been operated since 2003, and prior to that, shift workers worked to cover the entire day through three teams with three 8 h shifts. The shift work experience was confirmed using these questions: “Have you ever worked as a shift worker?” and “Are you currently working as a shift worker?” The response options for these questions were “yes” or “no”. Through these questions, all workers were classified into three groups such as current shift worker, past shift worker, and non-shift worker. Among current shift workers, the total duration of shift work was calculated by subtracting the year when shift work was started from the end of shift work.

### 2.4. Work-Related Injuries

Work-related injuries were confirmed using these question: “Have you ever been absent or treated for work-related injuries during the last 12 months?” The response options for these questions were “yes” or “no”.

### 2.5. Safety Health Score

The safety health score was developed through a research project in South Korea [25]. The score consists of 28 items on safety health awareness, education, and training for safety, information and communication for safety, and participation of safety health. Each item was scored from one to four points and summed. The scores were divided into three groups based on the total scores.

### 2.6. Statistical Analysis

Shift work experiences were used as exposure variables in our analysis. Before investigating the associations between shift work and work-related injuries, we explored distribution demographic characteristics by shift work experiences. Furthermore, we confirmed the prevalence of work-related injuries and identified differences in the prevalence of work-related injuries in demographic characteristics using the chi-square test.

In addition, we performed multiple logistic regression analysis to evaluate the associations between shift work and work-related injuries and estimated the odds ratio (OR) and 95% confidence intervals (CIs) using multiple logistic regression models. Furthermore, we conducted gender-stratified analyses to evaluate gender differences in shift work and work-related injuries. The model was adjusted for age, education level, marital status, alcohol consumption, smoking, level of safety health score, and place of work.

We conducted all statistical analyses using SAS version 9.4 (SAS Institute Inc., Cary, NC, USA) and considered two-tailed *p* values of <0.05 as significant.

## 3. Results

Among 13,610 workers enrolled in the present study, 8157 (59.9%) were current shift workers, 1820 (13.4%) were past shift workers, and 3633 (26.7%) were non-shift workers. Approximately, 80% of women were current shift workers. Current shift workers under the age of 30 were 73%. Approximately 80% of both fabrication (FAB) and back-end process workers were current shift workers. In addition, approximately 85% and 92% of engineers and operators, respectively, depending on the type of work, were working as current shift workers. On the other hand, over 70% of technicians and office workers did not work as shift workers. The distribution of data according to other major variables are shown in Table 1 and Table 2.

Table 3 shows the differences in the prevalence of work-related injuries with general characteristics of the study population. In this factory, the total prevalence of work-related injuries was 2.2%. The prevalence of work-related injuries in women (2.8%) was higher than that in men (1.7%). We observed a difference in prevalence based on education level, safety health score, place of work, type of work, and shift work experience (all *p*-values were <0.05). On the other hand, the prevalence of work-related injuries based on age and marital status differed only in men and in women according to smoking status. There was no difference in the prevalence of work-related injuries based on high-risk alcohol consumption in both men and women.

The association between shift work and work-related injuries are shown in Table 4. We found that current shift workers in the total population, in men, and in women were associated with a 2.7-, 2.3-, and 2.8-fold increased risk of work-related injuries, respectively. We did not observe statistically significant results in past shift workers. However, the risk of work-related injuries increased with a trend in the past and current shift workers compared with those who did not have experience of shift work. This trend effect on shift work and work-related injuries was statistically significant in both men and women (all *p*-values were <0.01).

Table 5 shows the results of the analyses performed to assess the association between work-related injuries and shift work durations in current shift workers. In the analyses of the total population, the risk of work-related injuries increased with longer shift work duration. When the analyses were stratified by gender, we found that shift work durations of 5–9 years, 10–14 years, and >15 years in women were associated with a 1.9-, 2.7-, and 3.9-fold increase in the risk of work-related injuries compared with the reference group (1–4 years), respectively. We confirmed that the increased risk of work-related injuries with longer shift work duration was stronger in women. In addition, these associations were only statistically significant in women workers (*p* = 0.03).

## 4. Discussion

We explored the association between the duration of shift work and work-related injuries among electronics factory workers under the rotating shift work system in South Korea. We found that the current shift workers presented a 2.65-fold higher risk of work-related injuries during last 12 months than non-shift workers. In addition, past shift workers showed a 1.71-fold higher risk of work-related injuries compared with non-shift workers. There was statistically significant dose-response relationship between shift work duration and work-related injuries among current shift workers. A gender-stratified model showed that the risk of work-related injury increased with shift work duration only in women.

According to the several previous studies, shift workers, compared with regular day workers, had an increased risk of work-related injuries. Four studies on rotating shift work included in this manuscript reported that workers were 1.21 to 1.97 times more prone to work-related injuries [18,26,27,28]. Furthermore, the workers who worked shifts over 8 h had a 1.32–1.98-fold higher risk of work-related injuries [27,28,29,30]. The study using the Korean Working Condition Survey of 2006 also reported that rotating shift work had a 1.79-fold higher risk of work-related injuries compared with non-shift work [31]. Our results also showed consistent findings of an association between shift work and work-related injuries through both domestic and international studies. However, the odds ratio of shift work on work-related injuries in our study was higher than that in previous studies—and this difference was more pronounced in women.

According to the systematic reviews of 14 relevant articles in 2011, shift workers, compared with regular day workers, had an increased risk of work-related injuries [9]. Four studies on rotating shift work included in this manuscript reported that workers were 1.21 to 1.97 times more prone to work-related injuries [18,26,27,28]. The study using the Korean Working Condition Survey of 2006 also reported that rotating shift work, compared with non-shift work, presented a 1.79-fold higher risk of work-related injuries. Our results also showed consistent findings of an association between shift work and work-related injuries through both domestic and international studies [10,11,31,32]. The odds ratio of shift work on work-related injuries in our study was higher than that in other studies. It could be that, in the electronics industry, compared with all other industries generally, the intensity of shift work is higher or that the durations of shift work are longer. Shift work is known to negatively affect safety and health issues in workplace. Therefore, it is important to find the best shift work schedule for workers. Although there are studies that rapid forward rotation is less harmful to worker’s health [33,34,35], the findings that deal with the best shift work schedules do not show consistent results. Moreover, most previous studies have not presented the intensity or duration of shift work of their study populations. Therefore, major considerations for the health protection of shift workers include reducing night work, providing less disruptive shift changes, and adjusting work load [36]. Further studies are needed to reveal the effects of intensity or duration of shift work on the association between shift work and work-related injuries. Studies that explore associations between workplace culture, tasks, and gender are also needed so as to determine the ideal shift schedule.

We also determined gender differences in work-related injuries with shift work duration among current shift workers. Our results are consistent with the findings of a recent study on a Canadian representative sample with a 6-year follow-up, which showed that women working non-standard shifts for a prolonged period had a greater risk of work-related injuries than men. Although we do not present this data in this article, the odds ratio for work-related injuries according to marital status was not statistically significant for either men or women. However, regardless of marriage, women are often the primary caregivers in their homes and spend more time than men caring for their children. As a result, women struggled to balance work and family and had less time for sleep after work. This study suggested that these factors increased the risk of work-related injuries in women [10,11]. Furthermore, in the factory where our research was conducted, women were known to work as operators who had a rest time only during meals, while men had more opportunities to rest. During the night shifts, men often worked as a technician or engineer who performed only two or three of maintenance work. This difference in job characteristics reflects the effects of shift work on work-related injuries. Another possible explanation is that the cumulative effect of shift work among current shift workers may be underestimated because the changes from a rotating shift to a day shift are more common in men than in women. There was similar dose-response relationships between shift work and work-related injuries, irrespective of gender, and the proportion of past shift workers was 18.2% for men and 8.1% for women (Table 3). Further research providing detailed information on shift work exposure and potential confounders is needed. Moreover, whatever the specific role of gender is in the association between shift work and work-related injuries, there is a need for ongoing research to prepare policies for gender equality in the workplace.

The notable findings in this study are that past shift workers, compared with day workers, showed a greater risk of work-related injuries and that, among current shift workers, the duration of shift work had a dose-response relationship with work-related injuries. There are several mechanisms that may explain the association between shift work and work-related injuries. First, fatigue due to insufficient sleep could influence shift workers’ cognitive function. Sleep disturbances may contribute to daytime sleepiness, an occurrence of fatigue, and an accumulation of fatigue, which in turn are risk factors for work-related injuries [9,27]. However, fatigue may not have long-term cumulative effects because it can be overcome by appropriate rest. Second, stressful conditions or depression caused by shift work could impact workers’ lack of cognitive function. A positively significant correlation was observed between shift work, job stress, work-life balance, and self-rated health. Therefore, workers with shift work are also those who struggle more with work-life balance and have more stress due to their jobs [37]. A third possible explanation is the disarrangement of the circadian rhythm. Shift work including night work leads to a disruption of the circadian rhythm. One study reported that shift workers have lower concentration scores than regular workers [38]. Other studies on health care workers have reported that shift workers have low levels of attention, concentration, and short-term memory [19,20]. Decline in attention and working memory is also known to be associated with sleep disturbances [39,40,41]. Therefore, a decrease in attention due to fatigue, depression, and circadian rhythm disruption can cause workers to make frequent mistakes and eventually lead to work-related injuries. The results of this study present a possibility that exposure to shift work had a cumulative effect on the risk of work-related injuries. Specifically, the results obtained in Table 3 and Table 4 imply that the effects of shift work contributing to the decline of cognitive function can be chronic or mental stress contributing workers’ lack of concentration. However, the above mechanism does not fully explain the findings in our study. We only confirmed that the duration of shift work is related to work-related injuries but could not confirm the mechanism underling the effects of shift work and work-related injuries in the current study. Further studies are needed to explore the detailed mechanism.

This study has several limitations. First, the causality cannot be explained due to the cross-sectional design of the study. However, the analysis was performed using a considerable number of samples, and our results were similar to those of previous studies. Second, the information on work-related injuries and characteristics of shift work were not an official record but self-reported data. In addition, we evaluated work-related injuries with simple questions, such as “Have you ever been absent or treated for work-related injuries during the last 1 year?” Therefore, we could not consider the severity of work-related injuries including loss of work and the degree of disability due to work-related injuries. Third, work-related injuries may change due to differences in the work environment. Furthermore, in the examined study, we could not consider factors such as level of supervision, specific type of tasks, and break time during shift work. Despite these limitations, we not only investigated shift work but also the duration of shift work. To obtain more precise results, we also conducted analysis with varying covariates, including type of task and safety health scores.

## 5. Conclusions

According to the results of this study, shift work was negatively associated with work-related injuries. Furthermore, work-related injuries were influenced by gender. Therefore, our study suggests that, along with shift work, the role of gender in the workplace or household should also be understood to effectively reduce work-related injuries. Since the present study suffers from limitations outlined above, further experimental and theoretical efforts are advisable on relevant topics such as the cause of injury, the severity of injury, and the culture of safety and health in the workplace. These studies work to clarify associations between shift work and work-related injuries.

## Figures and Tables

**Table 1:** Work characteristics of study population among electronics factory workers (N = 13,610).

			Total (N = 13,610)		
			Current Shift Worker	Past Shift Worker	Non-Shift Worker
		N (%)	N (%)	N (%)	N (%)
Total		13,610	8157	1820	3633
Place of work	FAB	5847 (43.0)	4503 (77.0)	846 (14.5)	498 (8.5)
	back-end process	3581 (26.3)	2909 (81.2)	364 (10.2)	308 (8.6)
	Laboratory	1498 (11.0)	204 (13.6)	168 (11.2)	1126 (75.2)
	Office	1065 (7.8)	73 (6.9)	118 (11.1)	874 (82.1)
	Others	1619 (11.9)	468 (28.9)	324 (20.0)	827 (51.1)
Type of work	Office worker	512 (3.8)	4 (0.8)	112 (21.9)	396 (77.3)
	Technician	3901 (28.7)	190 (4.9)	872 (22.4)	2839 (72.8)
	Engineer	2792 (20.5)	2381 (85.3)	321 (11.5)	90 (3.2)
	Operator	5780 (42.4)	5319 (92.0)	354 (6.1)	107 (1.9)
	Support	625 (4.6)	263 (42.1)	161 (25.8)	201 (32.2)
Location of factory	A	5268 (38.7)	3759 (71.4)	568 (10.8)	941 (17.8)
	B	8333 (61.2)	4398 (52.8)	1252 (15.0)	2683 (32.2)
	Others	9 (0.1)	0	0	9 (100.0)
Safety health score	T1 (34–79)	4580 (33.7)	2722 (59.4)	600 (13.1)	1258 (27.5)
	T2 (79–88)	4724 (34.7)	2956 (62.6)	574 (12.1)	1194 (25.3)
	T3 (88–112)	4306 (31.6)	2479 (57.6)	646 (15.0)	1181 (27.4)

The difference in the rate of each variable by shift work experience was statistically significant. (All *p*-values were <0.01 by chi-square test and Fisher’s exact test). FAB: fabrication.

**Table 2:** General characteristics of study population among electronics factory workers (N = 13,610).

			Total (N = 13,610)		
			Current Shift Worker	Past Shift Worker	Non-Shift Worker
		N (%)	N (%)	N (%)	N (%)
Total		13,610	8157	1820	3633
Gender	Men	7081 (52.0)	2798 (39.5)	1291 (18.2)	2992 (42.3)
	Women	6529 (48.0)	5359 (82.1)	529 (8.1)	641 (9.8)
Age	<30 years	5652 (41.5)	4143 (73.3)	313 (5.5)	1196 (21.2)
	30–40 years	6042 (44.4)	3574 (59.2)	800 (13.2)	1668 (27.6)
	≥40 years	1916 (14.1)	440 (23.0)	707 (36.9)	769 (40.1)
Educational level	High school diploma	4529 (33.3)	3988 (88.1)	423 (9.3)	118 (2.6)
	Graduated college	4504 (33.1)	3623 (80.4)	539 (12.0)	342 (7.6)
	Graduated university	3519 (25.8)	514 (14.6)	679 (19.3)	2326 (66.1)
	Higher than graduate school	1058 (7.8)	32 (3.0)	179 (16.9)	847 (80.1)
Marital status	Unmarried	6099 (44.8)	4144 (67.9)	433 (7.1)	1522 (25.0)
	Married or living common	7352 (54.0)	3894 (53.0)	1357 (18.5)	2101 (28.6)
	Others	159 (1.2)	119 (74.8)	30 (18.9)	10 (6.3)
Alcohol (≥2 times/week)	No	10,308 (75.7)	6370 (61.8)	1272 (12.3)	2666 (25.9)
	Yes	3302 (24.3)	1787 (54.1)	548 (16.6)	967 (29.3)
Smoking	Never	7747 (56.9)	5003 (64.6)	851 (11.0)	1893 (24.4)
	Ex-smoker	2672 (19.6)	1476 (55.2)	438 (16.4)	758 (28.4)
	Current smoker	3191 (23.5)	1678 (52.6)	531 (16.6)	982 (30.8)

The difference in the rate of each variable by shift work experience was statistically significant. (All *p*-values were <0.01 by chi-square test and Fisher’s exact test).

**Table 3:** The prevalence of self-reported work-related injuries in the previous year among electronics factory workers.

		Total	Prevalence of Work-Related Injuries	Men	Prevalence of Work-Related Injuries	Women	Prevalence of Work-Related Injuries
		N	%	N	%	N	%
Total		13,610	2.2	7081	1.7	6529	2.8
Place of work	FAB	5847	2.1	2861	1.9	2986	2.3
	back-end process	3581	3.5	1114	2.9	2467	3.7
	Laboratory	1498	1.5	1125	1.2	373	2.4
	Office	1065	0.9	772	0.5	293	1.7
	Others	1619	1.5	1209	1.1	410	2.7
	*p*-value		<0.01		<0.01		0.02
Type of work	Office worker	512	1.6	348	2.0	164	0.6
	Technician	3901	0.9	3377	0.8	524	1.2
	Engineer	2792	2.7	2792	2.7	0	0.0
	Operator	5780	3.0	0	0.0	5780	3.0
	Support	625	2.1	564	1.4	61	8.2
	*p*-value		<0.01		<0.01		<0.01
Location of factory	A	5268	1.9	2473	1.6	2795	2.2
	B	8333	2.4	4599	1.7	3734	3.4
	Others	9	0.0	9	0.0	0	0.0
	*p*-value		*0.13*		*0.91*		
Safety health score	T1 (34–79)	4580	3.0	2255	2.2	2325	3.9
	T2 (79–88)	4724	2.0	2365	1.8	2359	2.1
	T3 (88–112)	4306	1.7	2461	1.1	1845	2.5
	*p*-value		<0.01		<0.01		<0.01
Shift work	Current shift worker	8157	3.0	2798	2.6	5359	3.1
	Past shift work	1820	1.7	1291	1.5	529	2.1
	Non-shift work	3633	0.9	2992	0.8	641	1.1
	*p*-value		<0.01		<0.01		<0.01
Age	<30 years	5652	2.2	1717	0.8	3935	2.8
	30~<40 years	6042	2.5	3562	2.1	2480	3
	≥40 years	1916	1.5	1802	1.5	114	1.8
	*p*-value		0.04		<0.01		0.71
Educational level	High school diploma	4529	3.1	827	3.1	3702	3.1
	Graduated college	4504	2.6	2418	2.2	2086	3.1
	Graduated university	3519	0.9	2906	0.9	613	1.0
	Higher than graduate school	1058	1.4	930	1.3	128	2.3
	*p*-value		<0.01		<0.01		0.03
Marital status	Unmarried	6099	2.3	2355	1.2	3744	3.0
	Married or living common	7352	2.0	4666	1.8	2686	2.5
	Others	159	6.9	60	8.3	99	6.1
	*p*-value		<0.01		<0.01		0.07
Alcohol (≥2 times/week)	No	10,308	2.3	4992	1.8	5316	2.8
	Yes	3302	1.9	2089	1.2	1213	3.1
	*p*-value		0.22		0.08		0.51
Smoking	Never	7747	2.3	2666	1.8	5081	2.6
	Ex-smoker	2672	2.0	1900	1.4	772	3.4
	Current smoker	3191	2.2	2515	1.7	676	4.1
	*p*-value		0.61		0.65		0.05

All *p*-values were estimated with a chi-square test.

**Table 4:** Odds ratio for experience of shift work and self-reported work-related injuries in the previous year among electronics factory workers.

	Total				Men				Women			
	N	Case	Crude Model	Adjusted Model	N	Case	Crude Model	Adjusted Model	N	Case	Crude Model	Adjusted Model
			OR (95% CI)	OR (95% CI)			OR (95% CI)	OR (95% CI)			OR (95% CI)	OR (95% CI)
Total	13,610	303			7081	117			6529	186		
Non-shift work	3633	31	1 (Ref)	1 (Ref)	2992	24	1 (Ref)	1 (Ref)	641	7	1 (Ref)	1 (Ref)
Past shift work	1820	30	1.95 (1.18, 3.23)	1.71 (0.95, 3.06)	1291	19	1.85 (1.01, 3.38)	1.49 (0.73, 3.06)	529	11	1.92 (0.74, 5.00)	1.87 (0.64, 5.50)
Current shift work	8157	242	3.55 (2.44, 5.17)	2.65 (1.49, 4.70)	2798	74	3.36 (2.11, 5.34)	2.32 (1.11, 4.85)	5359	168	2.93 (1.37, 6.27)	2.79 (1.03, 7.60)
*p-*value				<0.01				<0.01				<0.01

Adjusted for age, education level, marital status, alcohol consumption, smoking, level of safety health score, and place of work. All *p*-values were estimated with logistic regression and multiple logistic regression.

**Table 5:** Odds ratio for duration of shift work and self-reported work-related injuries in the previous year among electronics factory workers.

	Total				Men				Women			
	N	Case	Crude Model	Adjusted Model	N	Case	Crude Model	Adjusted Model	N	Case	Crude Model	Adjusted Model
			OR (95% CI)	OR (95% CI)			OR (95% CI)	OR (95% CI)			OR (95% CI)	OR (95% CI)
Current shift worker	8080 ^¶^	239			2783 *	74			5297 ^§^	165		
1–4 years	1215	20	1 (Ref)	1 (Ref)	527	8	1 (Ref)	1 (Ref)	688	12	1 (Ref)	1 (Ref)
5–9 years	2430	72	1.82 (1.11, 3.01)	1.79 (1.07, 2.98)	575	15	1.74 (0.73, 4.13)	1.34 (0.45, 3.97)	1855	57	1.79 (0.95, 3.35)	1.91 (1.01, 3.62)
10–14 years	2761	93	2.08 (1.28, 3.39)	2.31 (1.32, 4.04)	947	32	2.27 (1.04, 4.96)	1.48 (0.42, 5.24)	1814	61	1.96 (1.05, 3.66)	2.66 (1.34, 5.25)
≥15 years	1674	54	1.99 (1.19, 3.35)	2.57 (1.31, 5.05)	734	19	1.72 (0.75, 3.97)	1.10 (0.28, 4.32)	940	35	2.18 (1.12, 4.23)	3.94 (1.68, 9.24)
*p-*value				0.01				0.19				0.03

Adjusted for age, education level, marital status, alcohol consumption, smoking, level of safety health score, and place of work. All *p*-values were estimated with logistic regression and multiple logistic regression. ^¶^ Exclusion of 77 cases unable to calculate shift work duration. * Exclusion of 15 cases unable to calculate shift work duration. ^§^ Exclusion of 62 cases unable to calculate shift work duration.

## Abbreviations

OR	odds ratio
CI	confidence interval
FAB	fabrication
